# Nitroglycerin can facilitate weaning of difficult-to-wean chronic obstructive pulmonary disease patients: a prospective interventional non-randomized study

**DOI:** 10.1186/cc9326

**Published:** 2010-11-15

**Authors:** Christina Routsi, Ioannis Stanopoulos, Epaminondas Zakynthinos, Panagiotis Politis, Vassilios Papas, Demetrios Zervakis, Spyros Zakynthinos

**Affiliations:** 1 Critical Care Department, Medical School of Athens University, Evangelismos Hospital, 45-47 Ipslilantou Str., Athens 106 76, Greece; 2 Respiratory Failure Unit, Aristotle University, G. Papanikolaou Hospital, Exohi, Thessaloniki 57 010, Greece

## Abstract

**Introduction:**

Both experimental and clinical data give convincing evidence to acute cardiac dysfunction as the origin or a cofactor of weaning failure in patients with chronic obstructive pulmonary disease. Therefore, treatment targeting the cardiovascular system might help the heart to tolerate more effectively the critical period of weaning. This study aims to assess the hemodynamic, respiratory and clinical effects of nitroglycerin infusion in difficult-to-wean patients with severe chronic obstructive pulmonary disease.

**Methods:**

Twelve difficult-to-wean (failed ≥ 3 consecutive trials) chronic obstructive pulmonary disease patients, who presented systemic arterial hypertension (systolic blood pressure ≥ 140mmHg) during weaning failure and had systemic and pulmonary artery catheters in place, participated in this prospective, interventional, non-randomized clinical trial. Patients were studied in two consecutive days, i.e., the first day without (Control day) and the second day with (Study day) nitroglycerin continuous intravenous infusion starting at the beginning of the spontaneous breathing trial, and titrated to maintain normal systolic blood pressure. Hemodynamic, oxygenation and respiratory measurements were performed on mechanical ventilation, and during a 2-hour T-piece spontaneous breathing trial. Primary endpoint was hemodynamic and respiratory effects of nitroglycerin infusion. Secondary endpoint was spontaneous breathing trial and extubation outcome.

**Results:**

Compared to mechanical ventilation, mean systemic arterial pressure, rate-pressure product, mean pulmonary arterial pressure, and pulmonary artery occlusion pressure increased [from (mean ± SD) 94 ± 14, 13708 ± 3166, 29.9 ± 4.8, and 14.8 ± 3.8 to 109 ± 20mmHg, 19856 ± 4877mmHg b/min, 41.6 ± 5.8mmHg, and 23.4 ± 7.4 mmHg, respectively], and mixed venous oxygen saturation decreased (from 75.7 ± 3.5 to 69.3 ± 7.5%) during failing trials on Control day, whereas they did not change on Study day. Venous admixture increased throughout the trial on both Control day and Study day, but this increase was lower on Study day. Whereas weaning failed in all patients on Control day, nitroglycerin administration on Study day enabled a successful spontaneous breathing trial and extubation in 92% and 88% of patients, respectively.

**Conclusions:**

In this clinical setting, nitroglycerin infusion can expedite the weaning by restoring weaning-induced cardiovascular compromise.

## Introduction

In patients with chronic obstructive pulmonary disease (COPD), the rate of weaning failure is high (>25%) and results in prolonged mechanical ventilation that increases both morbidity and mortality [1-4]. The most common pathophysiologic cause of unsuccessful weaning is thought to be failure of the respiratory muscle pump [5]. However, some difficult-to-wean COPD patients fail despite initial adequate ventilatory capacities. It has been suggested that the enormous workload that these patients face during weaning may result in cardiovascular distress and acute cardiac dysfunction [6].

Both experimental and clinical data give convincing evidence of acute cardiac dysfunction as the origin or a cofactor of weaning failure. Considerable negative intrathoracic pressures developed at inspiration during airway obstruction or pulmonary dynamic hyperinflation or both increase venous return (that is, preload) and also effectively increase left ventricular afterload [7,8]. Such increases may not be tolerated by spontaneously breathing patients with compromised heart function [7]. Patients with COPD have airway obstruction and commonly exhibit pulmonary dynamic hyperinflation [2-4], and recent data [9] show that COPD itself is a powerful independent risk factor for cardiovascular morbidity and mortality, suggesting that occult cardiac dysfunction could be frequent in patients with COPD. Indeed, cardiogenic pulmonary edema was developed during weaning of difficult-to-wean COPD patients with concomitant cardiovascular disease [10]. Furthermore, in potentially-able-to-wean COPD patients without obvious cardiac disease, a spontaneous breathing trial induced a significant left ventricular ejection fraction reduction not explained by a myocardial contractility decrease due to ischemia, thus implying a weaning-induced increase in afterload [11]. This increase in left ventricular afterload should be higher in patients demonstrating systemic arterial hypertension, which is quite frequent in COPD patients during weaning failure [12,13]. Therefore, it could be suggested that a treatment targeting the cardiovascular system might help the heart to tolerate the critical period of weaning more effectively. Vasodilators decrease the pressure gradients for venous return and right and left ventricular ejection and can affect left ventricular performance in a manner similar to that of the increased intrathoracic pressure [7]. To our knowledge, pharmaceutical interventions with such agents in COPD patients who fail weaning attempts have not been tested so far.

In the present study, we hypothesized that using nitroglycerin as the vasodilator agent to reduce venous return and right and left ventricular afterload could facilitate the weaning course in difficult-to-wean COPD patients. Accordingly, we studied the hemodynamic, respiratory, and clinical effects of nitroglycerin infusion during weaning of severe COPD patients exhibiting systemic arterial hypertension during repeatedly failing spontaneous breathing trials. The primary endpoint was hemodynamic and respiratory effects of nitroglycerin infusion. The secondary endpoint was spontaneous breathing trial and extubation outcome. Preliminary results of this study were presented at an international meeting [14].

## Materials and methods

### Patient selection

COPD patients who were intubated and mechanically ventilated because of acute decompensation in the intensive care unit of the Evangelismos Hospital, Athens, Greece, were considered eligible for the study. COPD was diagnosed on the basis of clinical history, blood gases, chest radiographic findings, and previous pulmonary function tests and hospital admissions. The appropriate institutional ethics committee approved the study, and informed written consent was obtained from each patient's close relative.

Inclusion criteria for study entry were the following: (a) The underlying cause of acute decompensation of COPD had resolved, and the primary physician had considered the patients ready to wean by performing spontaneous breathing trials. Criteria used in our institution for not attempting such spontaneous breathing trials [12] are similar to those of others [13]: known or suspected increased intracranial pressure, unstable coronary artery disease, heart rate of at least 120 beats per minute, positive end-expiratory pressure of greater than 5 cm H_2_O, pulse oximetric measurement of arterial oxygen saturation of less than 92%, fractional concentration of inspired oxygen (FiO_2_) of greater than 0.6, infusion of neuromuscular blocking drugs within the preceding 3 days, absent cough and gag reflex, or unresponsiveness to noxious stimuli. (b) Patients were difficult to wean; that is, they had failed at least three consecutive spontaneous breathing trials. (c) During spontaneous breathing trial failure, patients presented respiratory distress and systemic arterial hypertension, defined as systolic arterial blood pressure of at least 140 mm Hg [15]. (d) Systemic and pulmonary artery catheters inserted by the patients' physicians as part of patient management to support the weaning process were present. Exclusion criteria were previous home care ventilation, unconsciousness or need for sedation, and occurrence of an unstable coronary episode (acute myocardial infarction or unstable angina) and/or prior nitroglycerin use during current intensive care unit admission/stay. All consecutive patients fulfilling the criteria between January 2002 and February 2007 were included in the study. During this period, 52 patients with acute COPD decompensation requiring invasive mechanical ventilation were admitted to our center (2.6% of total admissions). Of these patients, 22 (42.3%) were difficult to wean, but only 12 patients fulfilled the criteria as 2 patients did not exhibit systemic arterial hypertension during spontaneous breathing trial failure, 2 patients had received nitroglycerin because of a coronary episode, and 6 patients did not have a pulmonary artery catheter in place during weaning. During the study, a physician not involved in the protocol was present to provide patient care.

### Measurements

The hemodynamic and gas exchange measurements and the calculations of hemodynamic and oxygenation variables were performed as previously described [12]. Correct positioning of the pulmonary artery catheter was verified by chest radiography, blood gas sampling, and waveform characteristics. The proximal and distal ports of the pulmonary artery catheter and the systemic artery catheter were connected to strain-gauge manometers that provided continuous measurements of right atrial, pulmonary, and systemic arterial pressures, respectively. Pulmonary artery occlusion pressure was measured after balloon inflation and wedging and was read at end-expiration. Cardiac output was determined by thermodilution using an Opti-Q pulmonary artery catheter connected to the Q-Vue continuous cardiac output computer (Abbott Laboratories, Abbott Park, IL, USA).

For gas exchange measurements, partial pressures of oxygen (PO_2_) and carbon dioxide (PCO_2_), pH, hemoglobin oxygen saturation (SO_2_), and hemoglobin concentration (Hb) were determined from blood samples anaerobically drawn from the arterial line and the distal port of the pulmonary artery catheter. Samples were immediately analyzed for blood gases (ABL 600; Radiometer Medical ApS, Brønshøj, Denmark).

The hemodynamic and gas exchange measurements and the calculations of hemodynamic and oxygenation variables were performed as previously described [12]. The rate-pressure product (RPP) was calculated as the heart rate times systolic arterial blood pressure [13].

Airway pressure was measured at the external end of the endotracheal or tracheostomy tube with a side port connected to a pressure transducer (Validyne MP-45, ± 100 cm H_2_O; Validyne Engineering Corp., Northridge, CA, USA). Distal to this side port, flow was measured with a heated pneumotachograph (Hans Rudolph, Inc., Kansas City, MO, USA). The pressure drop across the pneumotachograph was measured with a pressure transducer (Validyne MP-45, ± 2 cm H_2_O; Validyne Engineering Corp.). Volume was obtained by integration of the flow signal. Frequency was measured from the flow signal. During a temporal disconnection from the ventilator before the spontaneous breathing trial, maximum inspiratory pressure was measured as the maximal negative excursion in airway pressure during 20-second occlusion using a one-way valve [16]. The ratio of frequency to tidal volume (index of rapid shallow breathing) was calculated.

### Protocol

Patients were placed in semirecumbent position while they were ventilated in the assist-control mode with the ventilator settings prescribed by the primary physician. Patients then underwent a spontaneous breathing trial via a T-piece circuit while receiving the same FiO_2 _as during mechanical ventilation and gas humidification. Trials lasted for 2 hours unless patients met at an earlier time point; the criteria used to define spontaneous breathing trial failure were the following [17]: tachypnea (frequency of greater than 35 breaths per minute), arterial hemoglobin oxygen saturation (SaO_2_) of less than 85% to 90% on pulse oximetry, tachycardia (heart rate of greater than 120 to 140 beats per minute) or a sustained change in heart rate of more than 20%, systolic arterial blood pressure of greater than 180 to 200 mm Hg or less than 90 mm Hg, arrhythmias, increased accessory muscle use, diaphoresis, and onset or worsening of discomfort. The ability of the patient to remain free of these criteria at the end of the trial was defined as successful spontaneous breathing trial, and the patient was extubated. Extubation was defined as successful when spontaneous breathing was sustained for more than 48 consecutive hours after the T-piece trial, without development of any of the criteria of weaning failure. Patients who met these criteria during the 2-hour trial or within 48 hours after extubation were put back on assist-control mechanical ventilation, and the weaning was defined as spontaneous breathing trial failure or extubation failure, respectively. Weaning failure or success was judged by the primary physicians, who were not the study investigators. After resumption of mechanical ventilation, small-bolus infusions of propofol (0.5 to 1 mg/kg) were given if required in weaning failure patients to achieve synchronization with the ventilator, and patients were not disconnected from the ventilator for the subsequent 24 hours.

Patients were studied on two consecutive days: the first day without (Control day) and the second day with (Study day) nitroglycerin. Nitroglycerin was administered by continuous intravenous infusion starting at the beginning of the spontaneous breathing trial, and its dose was titrated to maintain normal systolic arterial blood pressure (that is, 120 to 139 mm Hg) [15]. Whenever the spontaneous breathing trial failed, administration of nitroglycerin was stopped at the time of resumption of mechanical ventilation. In case of trial success, nitroglycerin dose was gradually decreased and ceased during the subsequent hours, always titrated to systolic arterial blood pressure. No change in patients' treatment was made between Control day and Study day.

Twelve-lead electrocardiograph and arterial saturation were continuously recorded. Complete hemodynamic and oxygenation measurements were performed during mechanical ventilation, immediately before disconnection from the ventilator, and at 10 minutes (Start) and 2 hours (End) after the beginning of the spontaneous breathing trial. Breath components were also measured during mechanical ventilation and at 2 minutes (Start) and 2 hours (End) after disconnection from the ventilator. If the patient met the criteria of weaning failure before the end of the trial, all measurements were taken at the last minute of the trial (End).

### Statistical analysis

Data are reported as mean ± standard deviation. Distribution normality was tested by the Kolmogorov-Smirnov test. Comparison of data between mechanical ventilation and the start and end of the spontaneous breathing trial and between Control day and Study day was done by using two-way analysis of variance (ANOVA) with repeated measurements across time, followed by Scheffe test for *post hoc *comparisons. Differences between qualitative variables were assessed by Fisher exact test. A *P *value of less than 0.05 was considered statistically significant.

## Results

Twelve patients (9 men, 72 ± 7 years old) were included. Demographic and clinical characteristics of the patients are shown in Table 1. Patients had been 11 ± 6 days on mechanical ventilation and had difficult weaning with repeatedly failing weaning trials (6 ± 2). The causes of acute decompensation and respiratory failure were acute exacerbation (that is, an acute bout of pulmonary inflammation involving increased secretion of purulent sputum) in 9 patients, abdominal surgery in 2 patients, and gastrointestinal hemorrhage in 1 patient. Three patients had a history of intubation and mechanical ventilation. Eleven patients were on long-term domiciliary oxygen therapy. None was on home mechanical ventilation. Eleven of the patients had pulmonary function tests and blood gasses when stable in the months prior to admission, and their forced vital capacity was 55.2% ± 18.3% predicted, forced expiratory volume in one second was 27.4% ± 6.6% predicted, and partial pressure of arterial carbon dioxide was 47 ± 8 mm Hg. Echocardiography (transthoracic or transesophageal or both) performed during mechanical ventilation 1 to 2 days before study enrollment demonstrated mild to moderate left ventricular wall hypertrophy combined with diastolic dysfunction in 4 patients and left ventricular segmental wall motion abnormalities suggestive of previous inferior infarction in 3 patients [18]. Right ventricular free wall hypertrophy combined with right atrium and ventricle dilation, with increase in the ratio between right and left ventricular end diastolic volume but without modification of septum kinetics, was detected in 3 patients [19]. No severe valvular disease was demonstrated in any patient. Left ventricular ejection fraction was 58% ± 8% (range of 46% to 71%) (normal value of greater than 50%) [18,19]. At the time of the study, all patients were hemodynamically stable during mechanical ventilation without the use of any vasoactive agent. Sedation (propofol 2 to 4 mg/kg per hour) had stopped for at least 4 hours, and all patients had a Ramsay Sedation Scale level 2. Patients were ventilated in the assist-control mode with a Siemens 300 ventilator (Siemens, Solna, Sweden) through a cuffed endotracheal (*n *= 9) or tracheostomy (*n *= 3) tube and FiO_2 _of 0.4 to 0.5.

**Table 1:** Characteristics of the patients with chronic obstructive pulmonary disease

Patient	Days of MV	ET ID, mm	MIP, cm H_2_O	Cause of acute respiratory failure/Prior cardiovascular disease	Failed trial duration on Control day, minutes	Weaning trial outcome on Study day	Extubation outcome	ICU outcome
1	15	9	-20	GI hemorrhage/ Hypertension	10	S	S	A
2	11	8	-40	AAA repair/ Hypertension, CAD	30	S	S	A
3	9	8.5	-25	Acute exacerbation/ None	15	S	S	A
4	5	8.5	-30	Acute exacerbation/ CP	30	S	S	A
5	28	8^a^	-25	Acute exacerbation/ Hypertension, CP	110	S	NA	D
6	8	8.5^a^	-30	Acute exacerbation/ None	60	S	NA	A
7	9	8.5^a^	-28	Acute exacerbation/ CAD	45	S	NA	A
8	6	8	-30	Acute exacerbation/ CAD	110	S	S	A
9	10	8	-40	Acute exacerbation/ Hypertension, CP	60	S	S	A
10	9	8	-50	Acute exacerbation/ Hypertension	30	S	S	A
11	12	7.5	-22	Gastrectomy/ None	30	F	NA	A
12	10	8.5	-30	Acute exacerbation/ CAD	55	S	F	D

^a^, tracheostomy tube; A, alive; AAA, abdominal aortic aneurysm; CAD, coronary artery disease; CP, cor pulmonale; D, died; ET, endotracheal tube; F, failure; GI, gastrointestinal; ICU, intensive care unit; ID, internal diameter of tracheostomy tube; MIP, maximum inspiratory pressure; MV, mechanical ventilation; NA, not applicable; S, success.

On Control day, all patients met the criteria of weaning failure after 49 ± 33 minutes (Table 1) and were returned to mechanical ventilation. In contrast, on Study day, 11 out of 12 patients (92%) tolerated the spontaneous breathing trial (*P *< 0.001); one patient (number 11) failed this trial because of severe bronchospasm. Except for the 3 patients with a tracheostomy tube, patients who tolerated the spontaneous breathing trial (8 out of 11) were subsequently extubated. During the next 48 hours, 7 out of 8 extubated patients (88%) tolerated the spontaneous breathing without development of any of the criteria of weaning failure (extubation success), whereas 1 patient (number 12) met these criteria and was re-intubated (extubation failure). Therefore, 7 out of 9 endotracheally intubated patients who were potentially able to be extubated (that is, after excluding the 3 patients with tracheostomy) were finally extubated and weaned successfully (78% versus 0% without nitroglycerin infusion, *P *= 0.002). On Control day, 4 patients demonstrated new onset of electrocardiographic ischemic patterns, which were not detected on Study day. Ten of the 12 patients survived (Table 1) and were discharged from the intensive care unit; two patients died of sepsis and multi-organ failure. Two of the 10 patients who survived were discharged with ventilatory support. Nitroglycerin was given at a dose of 40 to 600 μg/minute. After the spontaneous breathing trial, nitroglycerin infusion was gradually reduced and then ceased after 20 hours in all patients.

### Hemodynamic variables

**Figure 1:**
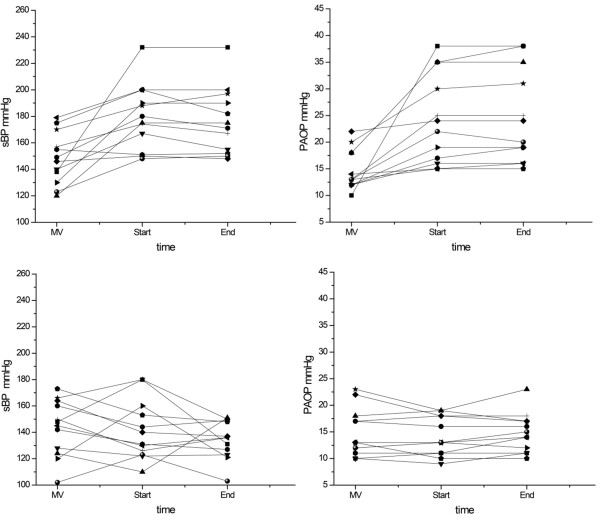
**Variations of systolic blood pressure (sBP) and pulmonary artery occlusion pressure (PAOP) during weaning trials**. Individual values of systolic blood pressure (sBP) (left) and pulmonary artery occlusion pressure (PAOP) (right) obtained on mechanical ventilation (MV) and at the 10th minute (Start) and last minute (End) of the spontaneous breathing trial on Control day (upper panel) and Study day (lower panel).

The effects of nitroglycerin on hemodynamics are shown in Table 2 and Figure 1. No significant difference in any of the hemodynamic variables was detected between the Control day and Study day during mechanical ventilation. From the start to the end of the spontaneous breathing trial, mean arterial blood pressure, RPP, mean pulmonary arterial pressure, and pulmonary artery occlusion pressure increased compared with mechanical ventilation on Control day but did not change on Study day (*P *= 0.002-*P *< 0.001, two-way ANOVA of the interaction day × time). Right atrial pressure was constantly lower during the trial on Study day compared with Control day (*P *= 0.001). Cardiac output increased during the trial on both Control day and Study day. Systemic vascular resistance did not change during the trial compared with mechanical ventilation on Control day but decreased on Study day. Similarly, pulmonary vascular resistance did not change during the trial on Control day but decreased at the end of the trial on Study day. Throughout the spontaneous breathing trial, right ventricular stroke work increased compared with mechanical ventilation on Control day but did not change on Study day (*P *= 0.07).

**Table 2:** Hemodynamics during weaning trials without (Control day) and with (Study day) nitroglycerin

Variable		Mechanical ventilation	Spontaneous breathing trial		*P *value^a^
				
			Start	End	
HR, beats/minute	Control day	91 ± 14	105 ± 14^b^	111 ± 15^b^	0.26
	Study day	94 ± 16	105 ± 16^b^	107 ± 13^b^	
sBP, mm Hg	Control day	149 ± 19	179 ± 26^c^	177 ± 25^c^	0.002
	Study day	144 ± 21	142 ± 23^d^	135 ± 15^d^	
mBP, mm Hg	Control day	94 ± 14	110 ± 21^b^	109 ± 20^c^	< 0.001
	Study day	91 ± 14	89 ± 15^d^	85 ± 12^d^	
RPP, mm Hg beats/minute	Control day	13,708 ± 3,166	19,041 ± 4,129^b^	19,856 ± 4,877^b^	< 0.001
	Study day	13,400 ± 2,637	14,738 ± 2,706^d^	14,625 ± 2,383^d^	
P_RA_, mm Hg	Control day	11.7 ± 3.2	13 ± 5.6	13.3 ± 5.8	0.001
	Study day	10.6 ± 2.9	8.3 ± 3.5^d^	8.7 ± 3.2^d^	
Mpap, mm Hg	Control day	29.9 ± 4.8	40.3 ± 6.2^b^	41.6 ± 5.8^b^	< 0.001
	Study day	28.8 ± 5.9	28.3 ± 4.6^d^	28.3 ± 3.9^d^	
Ppao, mm Hg	Control day	14.8 ± 3.8	23.3 ± 7.6^b^	23.4 ± 7.4^b^	< 0.001
	Study day	14.8 ± 4.9	14.2 ± 3.7^d^	14.8 ± 3.7^d^	
CO, L/minute	Control day	6.6 ± 2	8.2 ± 2	8.7 ± 2.5^c^	0.89
	Study day	6.4 ± 2	8.3 ± 2.3^c^	8.7 ± 2.7^b^	
SV, mL	Control day	73.2 ± 22.7	77.8 ± 16.3	78.8 ± 19.5	0.69
	Study day	70.3 ± 27.7	80.8 ± 26.8	81.3 ± 24.4	
SVR, dyne/s per cm^5^	Control day	1,084 ± 387	992 ± 300	931 ± 277	0.10
	Study day	1,085 ± 361	827 ± 268^c^	756 ± 252^b^	
PVR, dyne/s per cm^5^	Control day	199 ± 69	176 ± 70	171 ± 51	0.28
	Study day	192 ± 87	147 ± 57	137 ± 57^c^	
LVSW, g-m	Control day	78.2 ± 26.4	91.9 ± 31.2	93.4 ± 37.1	0.62
	Study day	73.5 ± 33.8	82.9 ± 35.6	78.3 ± 33.1	
RVSW, g-m	Control day	18.5 ± 9	28.8 ± 8.2^b^	30.8 ± 11.2^b^	0.07
	Study day	17.6 ± 10.3	22.8 ± 12.1	22.3 ± 10.1^d^	

Values are presented as mean ± standard deviation. ^a^*P *value of the interaction (day × time) of the repeated-measures two-way analysis of variance comparison between the Control day and Study day across time (that is, during mechanical ventilation and the 10th minute [Start] and last minute [End] of the spontaneous breathing trial). ^b^*P *< 0.01 versus mechanical ventilation. ^c^*P* < 0.05 versus mechanical ventilation. ^d^*P *< 0.05 versus control day. CO, cardiac output; HR, heart rate; LVSW, left ventricular stroke work; mBP mean arterial blood pressure; mPAP mean pulmonary arterial pressure; Ppao, pulmonary artery occlusion pressure; P_RA_, right atrial pressure; PVR, pulmonary vascular resistance; RPP, rate-pressure product; RVSW, right ventricular stroke work; sBP, systolic arterial blood pressure; SV, stroke volume; SVR systemic vascular resistance.

### Oxygenation

**Figure 2:**
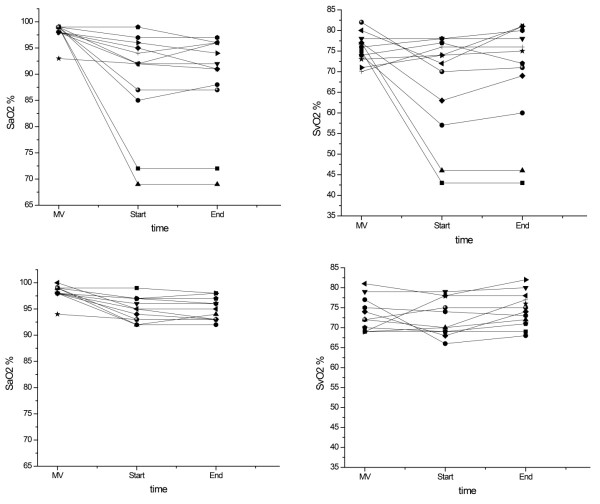
**Variations of arterial oxygen saturation (SaO_2_) and mixed venous oxygen saturation (SaO_2_) during weaning trials**. Individual values of arterial oxygen saturation (SaO_2_) (left) and mixed venous oxygen saturation (SvO_2_) (right) obtained on mechanical ventilation (MV) and at the 10th minute (Start) and last minute (End) of the spontaneous breathing trial on Control day (upper panel) and Study day (lower panel).

The effects of nitroglycerin on pulmonary and tissue oxygenation are presented in Table 3 and Figure 2. During mechanical ventilation, oxygenation variables were similar in Control day and Study day. During the spontaneous breathing trial, mixed venous oxygen saturation decreased compared with mechanical ventilation on Control day but did not change on Study day (*P *= 0.04). Venous admixture increased throughout the trial on both Control day and Study day, but the increase on Study day was lower (*P *= 0.04). Oxygen extraction ratio was similar during the spontaneous breathing trial on Control day and Study day.

**Table 3:** Breath components and oxygenation during weaning trials without (Control day) and with (Study day) nitroglycerin

Variable		Mechanical ventilation	Spontaneous breathing trial		*P *value^a^
				
			Start	End	
V_T_, Ml	Control day	617 ± 103	289 ± 123^b^	298 ± 114^b^	0.18
	Study day	608 ± 88	343 ± 114^b^	365 ± 73^b^	
Frequency, beats/minute	Control day	16 ± 3.8	29 ± 7.7^b^	29 ± 7.8^b^	0.44
	Study day	16 ± 4.1	25 ± 5^b^	26 ± 5^b^	
V_E_, L/minute	Control day	9.7 ± 2.9	8.1 ± 3.6	8.3 ± 3.3	0.51
	Study day	9.7 ± 3.2	8.8 ± 3.3	9.4 ± 2.6	
f/V_T_, beats/minute per liter	Control day	26 ± 8	114 ± 48^b^	108 ± 42^b^	0.03
	Study day	27 ± 7	82 ± 29^b,c^	73 ± 19^b,c^	
PaO_2_, mm Hg	Control day	130 ± 35	65 ± 18^b^	66 ± 17^b^	0.23
	Study day	132 ± 35	85 ± 18^c,d^	89 ± 23^c,d^	
PaCO_2_, mm Hg	Control day	51 ± 10	69 ± 13^b^	70 ± 13^b^	0.20
	Study day	49 ± 11	61 ± 13^b,c^	64 ± 16^b^	
pHa	Control day	7.41 ± 0.06	7.32 ± 0.07^b^	7.30 ± 0.07^b^	0.20
	Study day	7.42 ± 0.05	7.34 ± 0.06^d^	7.34 ± 0.07^b^	
SaO_2_, percentage	Control day	98.3 ± 1.6	89.2 ± 6.2^b^	88.8 ± 4.7^b^	0.01
	Study day	98.2 ± 1.5	95.3 ± 2.2^c^	94.9 ± 2.2^c^	
SvO_2_, percentage	Control day	75.7 ± 3.5	67.3 ± 7.2^d^	69.3 ± 7.5^d^	0.04
	Study day	73 ± 4.1	72.3 ± 4.3^c^	74.6 ± 4.3^c^	
Qs/Qt, percentage	Control day	15.0 ± 6.0	37.8 ± 17.8^b^	39.5 ± 13.8^b^	0.04
	Study day	14.3 ± 5.9	25.7 ± 6.6^c^	28.5 ± 8.1^d^	
O_2_ER, percentage	Control day	24.4 ± 3.3	24.7 ± 8.4	22.9 ± 8.5	0.32
	Study day	27 ± 4.1	24.8 ± 4.4	22.1 ± 4.5	

Values are presented as mean ± standard deviation. ^a^*P *value of the interaction (day × time) of the repeated-measures two-way analysis of variance comparison between the Control day and Study day across time (that is, during mechanical ventilation, the second minute [for breath components] or 10th minute [for oxygenation variables] [Start], and the last minute [End] of the spontaneous breathing trial). ^b^*P *< 0.001 versus mechanical ventilation. ^c^*P *< 0.05 versus control day. ^d^*P *< 0.05 versus mechanical ventilation. f/V_T_, frequency/tidal volume (index of rapid shallow breathing); O_2_ER, oxygen extraction ratio; PaCO_2_, arterial carbon dioxide partial pressure; PaO_2_, arterial oxygen partial pressure; pHa, arterial pH; Qs/Qt, venous admixture; SaO_2_, arterial oxygen saturation; SvO_2_, mixed venous oxygen saturation; V_E_, minute ventilation; V_T_, tidal volume.

### Pattern of breathing

Breath components are demonstrated in Table 3. No difference in any of the breath components was detected between Control day and Study day during mechanical ventilation. Tidal volume decreased and frequency increased throughout the trial compared with mechanical ventilation on both Control day and Study day. Index of rapid shallow breathing increased during the trial on both Control day and Study day, but the increase on Study day was lower (*P *= 0.03).

### Adherence to protocol

The target of normal systolic arterial blood pressure on Study day was achieved only partly in some patients and their systolic blood pressure intermittently remained higher than normal.

## Discussion

The present study performed in difficult-to-wean COPD patients exhibiting systemic arterial hypertension during repeatedly failing spontaneous breathing trials had the following main findings: (a) systemic arterial pressure, RPP, mean pulmonary arterial pressure, pulmonary artery occlusion pressure, and right ventricular stroke work increased and mixed venous oxygen saturation decreased during failing trials, whereas nitroglycerin infusion restored these changes; and (b) nitroglycerin administration enabled a successful spontaneous breathing trial and extubation in 92% and 88% of patients, respectively.

Our results suggest that, in the clinical setting of the present study, the use of nitroglycerin directed toward the cardiovascular system can expedite weaning, presumably by alleviating acute cardiac dysfunction. Weaning-induced acute cardiac dysfunction resulting in acute pulmonary congestion is a known cause or cofactor of weaning failure in predisposed COPD patients, particularly in those with pre-existing cardiac disease [10,13]; its mechanisms of development are complex and include increases in venous return and left ventricular preload, myocardial ischemia, diastolic left ventricular dysfunction and an increase in left ventricular afterload [10,13,20]. By restoring these mechanisms of development of acute cardiac dysfunction, nitroglycerine administration proved to facilitate the weaning of our patients. Indeed, 4 out of 12 patients demonstrated new onset of electrocardiographic ischemic patterns on Control day, and these patterns were not detected during nitroglycerin administration on Study day. Weaning increases myocardial oxygen demand by increasing sympathetic activity, work of breathing, and left ventricular afterload [7,8,10,11,13,20], thus inducing myocardial ischemia in the setting of pre-existing coronary artery disease [10,13,20]. A history of coronary artery disease was present in 4 of our 12 patients (Table 1), and 3 of the 4 had findings of infarction on rest echocardiography. As recent data suggest a significant association between COPD and coronary artery disease [9], several other patients of ours, particularly those with a history of hypertension, had an increased likelihood of occult ischemic heart disease [13] (Table 1). In our study, RPP, a global index of myocardial workload and oxygen demand [21], increased during failing trials on Control day, whereas nitroglycerin infusion restored this change, thus suggesting a beneficial effect of nitroglycerin on reducing myocardial oxygen demand and weaning-induced myocardial ischemia.

Abnormal left ventricular diastolic function has been reported frequently in COPD patients and may be related to coexisting hypertension and left ventricular hypertrophy and/or cardiac ischemia [22]. Potential causes of acute pulmonary congestion during weaning in patients with diastolic left ventricular dysfunction include the weaning-induced increases in venous return and left ventricular afterload, hypoxia, and tachycardia [23]. In our study, 5 out of 12 patients had a history of hypertension (Table 1) and 4 of the 5 demonstrated left ventricular wall hypertrophy on rest echocardiography. During failing weaning trials on Control day, these patients as well as patients exhibiting myocardial ischemia may have developed acute worsening of diastolic left ventricular dysfunction with a consequent increase in pulmonary artery occlusion pressure. By decreasing systemic arterial pressure, left ventricular afterload, and venous return and by preventing myocardial ischemia and acute deterioration of diastolic left ventricular dysfunction, nitroglycerine infusion should have avoided the increase in pulmonary artery occlusion pressure.

**Figure 3:**
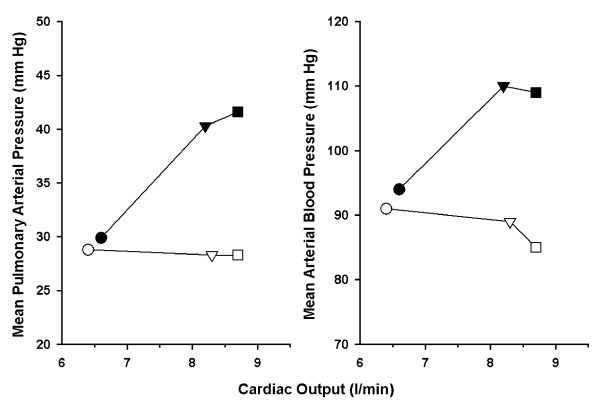
**Mean pulmonary arterial pressure and mean arterial blood pressure versus cardiac output during mechanical ventilation (circles) and at the start (triangles) and end (squares) of spontaneous breathing trials on Control day (closed symbols) and Study day (open symbols)**. Substantial increases in mean pulmonary arterial pressure and mean arterial blood pressure on Control day were cancelled by nitroglycerin infusion on Study day and a similar increase in cardiac output occurred on both days, strongly suggesting that nitroglycerin infusion on Study day abandoned the increased right and left ventricular afterload on Control day.

Another type of left ventricular diastolic dysfunction in COPD patients is due to interventricular dependence [24]. In COPD patients with pre-existing right ventricular disease associated with chronic pulmonary hypertension, weaning-induced increases in right ventricular afterload and stress may occur because of hypoxemia, hypercapnia combined with respiratory acidosis, and worsening of intrinsic positive end-expiratory pressure [10,25,26]. This phenomenon, together with a simultaneous increase in venous return, may lead to a marked right ventricular enlargement during weaning, thus impeding the diastolic filling of the left ventricle through an interventricular dependence mechanism [10,25]. In our study, most of the patients already met World Health Organization criteria for pulmonary hypertension (mean pulmonary arterial pressure of greater than 25 mm Hg) during mechanical ventilation (Table 2). The fact that a substantial increase in mean pulmonary arterial pressure on Control day was cancelled by nitroglycerin infusion on Study day and a similar increase in cardiac output occurred on both days (Figure 3) strongly suggests that nitroglycerin infusion on Study day abandoned the increased right ventricular afterload on Control day [26]; attenuation of the increased right ventricular stroke work by nitroglycerine has a similar meaning (that is, decrease of the weaning-induced increases in right ventricular afterload and stress). By reducing the weaning-induced increases in right ventricular afterload and venous return, nitroglycerin infusion may have averted a noticeable right ventricular enlargement and acute left ventricular diastolic dysfunction through interventricular dependence, thus contributing to the decrease of the weaning-induced increase in pulmonary artery occlusion pressure.

To the best of our knowledge, this is the first study in which nitrates were given as vasodilator therapy in difficult-to-wean COPD patients in order to expedite the weaning. However, nitroglycerin has been tested previously in stable or mechanically ventilated COPD patients with chronic pulmonary hypertension [27-29]; in accordance with our findings, nitroglycerin decreased mean pulmonary arterial pressure in stable mechanically ventilated COPD patients [28] and decreased both mean pulmonary arterial pressure and right ventricular stroke work in mechanically ventilated COPD patients [29]. In a recent anecdotal report, another type of vasodilator therapy (that is, phosphodiesterase 5 inhibitors [sildenafil]) was used to facilitate weaning in three difficult-to-wean COPD patients [30].

Potential limitations of the present study should be pointed out. First, our population was highly selected: the patients should have failed at least three consecutive spontaneous breathing trials and demonstrated systemic arterial hypertension during weaning failure before being included in the study. This may have accounted for the cardiovascular origin of or contribution to weaning failure in our patients. Therefore, the message of our study is not that the weaning failure in COPD patients is primarily or necessarily related to cardiovascular problems. Second, the number of patients studied is relatively small, despite the 5-year study duration and inclusion of consecutive patients; nowadays, only limited numbers of COPD patients require invasive mechanical ventilation since acute decompensation is frequently managed successfully by non-invasive mechanical ventilation, and our strict inclusion criteria resulted in high patient selection. However, each patient was studied twice, thus serving as his or her own control, and hemodynamic and respiratory responses to discontinuation of mechanical ventilation either with or without nitroglycerin infusion were homogeneous as indicated by the small standard deviations and the normal distribution of most continuous variables that allowed use of parametric statistical tests. Third, a non-randomized design was applied and potentially this could have resulted in an order effect. Indeed, it cannot be excluded that the hemodynamic and outcome results were related not only to the administration of nitrates but also to the fact that the Study day was 24 hours after the Control day. Fourth, we did not measure esophageal pressure to assess a transmural value of pulmonary artery occlusion pressure, which could be lower than that at end-expiration in patients actively contracting their expiratory muscles during expiration. Indeed, in four patients, the pulmonary artery occlusion pressure was increased on mechanical ventilation before the start of the spontaneous breathing trial. In these patients, the attending physicians considered that hyperinflation or active expiration or both contributed to this increase.

## Conclusions

The present non-randomized study performed in difficult-to-wean COPD patients exhibiting systemic arterial hypertension during failing spontaneous breathing trials demonstrated that nitroglycerin infusion can expedite the weaning, most likely by restoring the weaning-induced increases in venous return and left ventricular preload, myocardial ischemia, diastolic left ventricular dysfunction and the increase in left ventricular afterload, thus alleviating the weaning-induced acute cardiac dysfunction. However, because of high patient selection, the message of this study is not that weaning failure in COPD patients is primarily or necessarily related to weaning-induced acute cardiovascular problems.

## Key messages

• Nitroglycerin infusion can expedite the weaning in difficult-to-wean chronic obstructive pulmonary disease (COPD) patients exhibiting systemic arterial hypertension during failing spontaneous breathing trials.

• Nitroglycerin infusion most likely works by alleviating the weaning-induced acute cardiac dysfunction.

• Because of high patient selection, the message of this study is not that weaning failure in COPD patients is primarily or necessarily related to weaning-induced acute cardiovascular problems.

## Abbreviations

ANOVA: analysis of variance; COPD: chronic obstructive pulmonary disease; FiO_2_: fractional concentration of inspired oxygen; RPP: rate-pressure product.

## Competing interests

The authors declare that they have no competing interests.

## Authors' contributions

CR recruited patients, made measurements in patients, participated in the design of the study, interpreted the results, drafted the manuscript, and presented the findings at conferences. IS recruited patients, made measurements in patients, and participated in the design of the study. EZ performed echocardiographic studies, interpreted the results, and reviewed the manuscript. PP performed echocardiographic studies and reviewed the manuscript. VP and DZ made measurements in patients and reviewed the manuscript. SZ interpreted the results, provided statistical analysis, and wrote the final version of the manuscript. All authors read and approved the final manuscript.
